# Biocontrol of invasive pheretimoid earthworms using *Beauveria bassiana*

**DOI:** 10.7717/peerj.11101

**Published:** 2021-04-07

**Authors:** Maryam Nouri-Aiin, Josef H. Görres

**Affiliations:** Plant and Soil Science, University of Vermont, Burlington, VT, USA

**Keywords:** Amynthas, *B. bassiand*, Biocontrol, Invasive species, Pheretimoid, Invasive earthworms

## Abstract

**Background:**

Invasive species cause enormous costs of over $120 billion to the U.S. economy. Among biological invasions, the invasion by pheretimoid earthworms has gone relatively unnoticed and their invasion imposes yet unknown damage on USA agriculture and horticulture. The main dispersal is with horticultural goods such as plant material and composts. Pheretimoids affect commercially important hardwood forest. With no chemical agents currently certified for earthworm control nor any best horticultural practices, slowing the invasion is difficult.

**Methods:**

In this study we measured the efficacy of a commercial entomopathogenic fungal isolate of *B. bassiana* (BotaniGard^®^) to kill pheretimoid earthworms under greenhouse conditions. Four treatments of *B. bassiana* were applied: The commercial product as per label, re-cultured commercial *B. bassiana*, 15 g and 25 g millet grains mycotized with recultured product. In all, three bioassays were conducted in 2 consecutive years with two batches of BotaniGard^®^.

**Results:**

With fresh batches, all *B. bassiana* treatments with re-cultured product resulted in greater than 70% mortality within 4 weeks. Mortality was less than 60% when BotaniGard^®^ was used as prescribed by the label. When using 1-year old spores (refrigerated at 4 °C), mortality rates for *B. bassiana* treatments were less than 20% and not significantly different from the controls. However, *B. bassiana* still affected the earthworms by slowing their development from juvenile to adult stage.

**Conclusion:**

*B. bassiana* was effective against pheretimoid earthworms. Overall, mycotized millet grains did not significantly increase mortality over the re-cultured, directly applied *B. bassiana* spores. More experimentation is needed to find the mode of action of the re-cultured *B. bassiana* before investigating ways to improve the efficacy of *B. bassiana* when applied as prescribed on the label.

## Introduction

The damage and economic losses in agriculture and forestry associated with biological invasions in the United State are enormous (Office of Technology Assessment, 1993; Pimentel, Zuniga & Morrison, 2005). Even with phytosanitary regulations, introductions are still common (Pyšek et al., 2020). Most recognized among invasive organisms are weedy plants and arthropod pests (Kinahan et al., 2020; Kostivkovsky & Young, 2000; Pimentel, 2009). Below-ground invaders such as exotic earthworms go largely undetected or are not recognized as such.

Although there are several native North American earthworms’ species in northern North America (McCay et al., 2017), most invasive species were introduced from Europe (Gates, 1982) and Asia (Kinberg, 1867). A second wave of invasions by Asian earthworms, collectively named “pheretimoids”, is currently in progress in North America (Chang, Szlavecz & Buyer, 2016). The monetary cost of earthworm invasions has not been quantified probably because of the cryptic nature of the invasion and the “social standing” of earthworms. Earthworms are perceived as positive organisms in gardens and agriculture.

In New England, pheretimoids spread from horticultural operations into forest ecosystems (Görres, Bellitürk & Melnichuk, 2016) and have been present for approximately 70 years (Gates, 1954). But it has only been over the past three decades that they have invaded economically important northern hardwood forests, potentially damaging the forests that sustain the maple sirup industry. Pheretimoids have become a leading ecological concern in hardwood forests in the northern USA (Reynolds, 2015; Görres & Melnichuk, 2012; Migge-Kleian et al., 2006). Of the 16 pheretimoid species known to be present in North America (Chang, Szlavecz & Buyer, 2016), three frequently co-invade in North America: *Amynthas agrestis*, *A. tokioensis* and *Metaphire hilgendorfi* (Chang et al., 2018). The northerly expansion (Moore, Görres & Reynolds, 2018; Görres et al., 2018) of the three co-invading pheretimoid species may be due to the lengthening of the growing season in the past 30 years (Hayhoe et al., 2007; Bohlen et al., 2004). Although the invasive earthworm research focus has been on forested ecosystem, damage has been reported from nurseries and greenhouses, where the worms invade pots and production beds. Typically, the plants are deprived of medium around their roots causing drought symptoms.

Plant and compost products are likely vectors of the expansion of pheretimoid earthworms into the wild. A survey of Master Gardeners showed wide distribution of these earthworms in New England gardens (Bellitürk et al., 2015). In Connecticut, Vermont and New Hampshire 88%, 20% and 24% of respondents, respectively, stated that these earthworms were present in their gardens or nearby ecosystems. Midwest states are facing similar concerns regarding the distribution of pheretimoid earthworms (TOEROEK Associates Inc, 2015). The U.S. states of Wisconsin, California and New York (Wisconsin Department of Natural Resources (WDNR), 2009; California Department of Food and Agriculture (CDFA), 2017; New York Department of Environmental Conservation (NYDEC), 2014) have declared these species invasive or at least of concern with regulatory consequences for the horticultural industry. However, there are currently no realistic and legal control mechanisms, leaving the industry unprepared for complying with regulations. Vermicides previously used and expellants (such as mercury chloride or Mowrah meal) are no longer certified; more recently used organic treatments and chemical pesticides are off-label or their use prohibited (Potter et al., 2010; Potter, Redmond & Williams, 2011). To our knowledge, there are no biological controls of pheretimoids (McCay et al., 2020) and commercially available microbial insecticides such as *Bacillus thuringiensis* are not effective against earthworms (Saxena & Stotzky, 2001). However, Edwards & Fletcher (1988), showed that several fungi and some bacteria kill red wigglers, *Eisenia fetida*, and some fungi may infect earthworm cocoons (Nuutinen et al., 1991).

With some US states regulating pheretimoids in horticulture, environmentally friendly, easy to use methods that are acceptable in horticulture, must be found. Three co-occurring *Amynthas* species in Vermont are considered epi-endogeic, they feed on organic matter and usually are found in topsoil and beneath and in the leaf litter (Bouché, 1977). The epi-endogeic nature of pheretimoids suggest that their habitat intersects with that of entomopathogenic fungi which inhabit soil and leaf litter in nature (Samson, Parker & Jones, 1988; Spatafora & Blackwell, 1993). Several of these are available as commercial products. For example, *Beauveria bassiana* (Balsamo) Vuillemin in wettable powder formulation has been widely used for the control of many insect pests afflicting horticultural crops in North America. To test the efficacy of *B. bassiana* on pheretimoid earthworms, three greenhouse assays were conducted with both spore suspensions and mycotized millet grains.

## Materials and Methods

### Specimen collection

Juvenile pheretimoids were collected at the University of Vermont’s Centennial Woods (CW) Natural Area (44° 28′ 36″ N, 73° 11′ 12″ W) in June of 2018 and May and July 2019. At these times pheretimoids are still in their juvenile stage and cannot be identified to species by morphology. In July 2019, the earthworms were fully extended but still had not developed a clitellum. At this site the earthworm community is exclusively pheretimoids (Keller, Görres & Schall, 2017), they were also identified by their trashing behavior when picked up which is unique to these taxa in Vermont.

The collection site was on alluvial, poorly drained Limerick silt loam (coarse-silty, mixed, super active, nonacid, mesic Fluvaquentic Endoaquepts) (Soil Survey Staff, 2019). The canopy comprised white pine (*Pinus strobus* Linnaeus), hemlock (*Tsuga canadensis* (L.) Carriere), and sugar maple (*Acer saccharum* Marsh). The understory at the site was dominated by jewel weed (*Impatiens capensis* Meerb.). The likely local origin of the earthworms is a housing development with ornamental and vegetable gardens some 250 m up-slope from the study site where the presence of *A. agrestis*, *A. tokioensis* and *M. hilgendorfi* was confirmed. Collections from CW for another project indicated that *A. agrestis* was dominant there accounting for 95% of specimens (Nouri-Aiin & Görres, 2019; Keller, Görres & Schall, 2017). In 2018, haphazard collections obtained exclusively *A. agrestis*. However, in the second year of this study, collections for bioassays also yielded large numbers of *A. tokioensis*.

### Fungal isolate and preparation of liquid culture

We cultured pure *B. bassiana* from the commercial product (BotaniGard^®^ ES, GHM; BioWorks, Victor, NY, USA) on Potato Dextrose Agar (PDA, OXOID, Basingstoke, UK) in the dark at 23 ± 2 °C. Liquid culture inoculate was prepared, according to a protocol developed by Kim et al. (2014), by adding a conidial suspension (adjusted to 1 × 10^8^ conidia mL^−1^ in a 250 mL flask) of *B. bassiana* from the PDA to one-quarter-strength Sabouraud dextrose broth (1/4 SDB; Difco™, Bordeaux, France). Flasks were held on a rotary shaker (150 rpm) at 24 ± 2 °C for 3 days. The treatment strength of 10^8^ conidia per mL was obtained by serial dilution.

### Preparation of mycotized millet grains

We mycotized organic whole millet (*Panicum miliaceum* L.) grains (Healthy Living, South Burlington, Vermont) using a method modified from Hua & Feng (2005) and Bartlett & Jaronski (1988). Millet grains were selected as substrate for granular fungal formulation for two major reasons: it is a standard substrate for *B. bassiana* (Hua & Feng, 2005; Saranraj & Javaprakash, 2017) and it attracts earthworms as a food source (M. Nouri-Aiin and J. Görres, 2019, personal observation). Dry millet grains (250 g) were rinsed with distilled water and placed in autoclavable polyvinyl bags and soaked in 125 mL of water containing citric acid (0.4 mL^−1^). All bags were flattened in a tray and autoclaved at 121 °C for 40 min and cooled to ambient temperature. Each bag of autoclaved grains was inoculated with a homogenized mixture of 5 mL of liquid culture broth and held for 14 days at 23 ± 2 °C and a 16:8 h L:D photoperiod. After 10 days when millet grains were entirely covered by *B. bassiana*, the bags were opened at ambient temperature until a moisture content of <5% was reached.

Mycotized millet grains were refrigerated at 4 °C in the dark until use. All batches of mycotized millet grains were assessed for conidial concentration with a Neubauer hemocytometer. To determine germination rate for each bag, four plates of Sabouraud dextrose agar (30 g in 1 L water) were inoculated with 0.1 mL of the conidial suspension and held in the dark at 25 °C for 24 h. Conidia with germination tubes longer than their width were considered germinated by inspecting 400 conidia per Petri dish (Hywel-Jones & Gillespie, 1990). Conidial yield was determined with a Neubauer hemocytometer to quantify the number of conidia per gram. Three batches with germination rates >98% and ≈ 1 × 10^8^ conidia per gram were used in the bioassays. It was not possible to get accurate estimates of the conidia count and germination rate because of the inert ingredients.

### Experimental design

Three mesocosm bioassays were conducted to test the effect of the entomopathogenic fungus *Beauvaria bassiana* (BotaniGard^®^) on juveniles of *Amynthas* spp. The first was conducted in the spring of 2018, the second and third in the spring and summer of 2019, respectively. The assays in the spring of 2018 and 2019 were conducted with the same batch of a BotaniGard^®^ obtained in April 2018. These two assays differed only in the age of the BotaniGard^®^. For both of these assays fresh cultures were prepared as described above and the age of the juveniles was comparable. For assay 3 in the summer 2019, fresh product was purchased 4 weeks before the trial and, in this aspect, it resembled the first bioassay. However, the age of the juveniles was more mature in the summer 2019 assay. The bioassays were distributed over 2 years because these earthworms are annual species and develop fast so that similar life stages had to be collected over 2 years.

The treatments used in all trials included two controls (water, 15 g millet) and the following three treatments: suspension of pure *B. bassiana* cultured from the commercial product, 15 g and 25 g of mycotized millet (Table 1). To have a paired control for each treatment, an additional control of 25 g millet was added in the 2019 trials. In the June 2019 trial and the August 2019 trial, we added BotaniGard^®^ suspension prepared according to the label (1 × 10^12^ spores/mL). Treatments and controls were each replicated 15 times. The suspensions were applied directly to the earthworms before adding them to the pots. Millet was added to the growing medium prior to the earthworms.

**Table 1:** Treatment combinations and other parameters of the bioassays.

Assays	Assay 1	Assay 2	Assay 3
Assay Date	June 2018	June 2019 I	August 2019 II
Trial Length	5 weeks	5 weeks	4 weeks
Life Stage	juvenile	juvenile	mature juvenile
Date of BotaniGard^®^ Purchase	April 2018	April 2018	July 2019
Age of BotaniGard^®^	2 Months	14 Months	1 Month
**Treatment Characteristics**			
Water	**√**	**√**	**√**
Millet 15 g	**√**	**√**	**√**
Millet 25 g		**√**	**√**
*B. bassiana* 10^8^ conidia/mL	**√**	**√**	**√**
Mycoticized millet 15 g	**√**	**√**	**√**
Mycoticized millet 25 g	**√**	**√**	**√**
BotaniGard^®^ 10^12^ conidia/mL			**√**

Experimental units were prepared using 1-gallon pots lined with zippered, fine-meshed laundry bags 11 × 15 inch (Aspire, LADB-EA70339 ordered through Opentip.com) to prevent worms from escaping. To each bag were added 1,000 g of organic potting mix (All Purpose Mix, Promix, Quakertown, PA, USA) and seven non-clitellated (juveniles) pheretimoid earthworms. The pots were then arranged on a growing bench at the center of a greenhouse. They were watered twice a week until the pots leaked leachate. In 2018, the earthworms were enumerated at the end of a 5-week period. In the summer of 2019, earthworms were counted every 3 to 4 days during the experimental period in order to calculate LT_50_.

### Analysis of mortality

**Figure 2:**
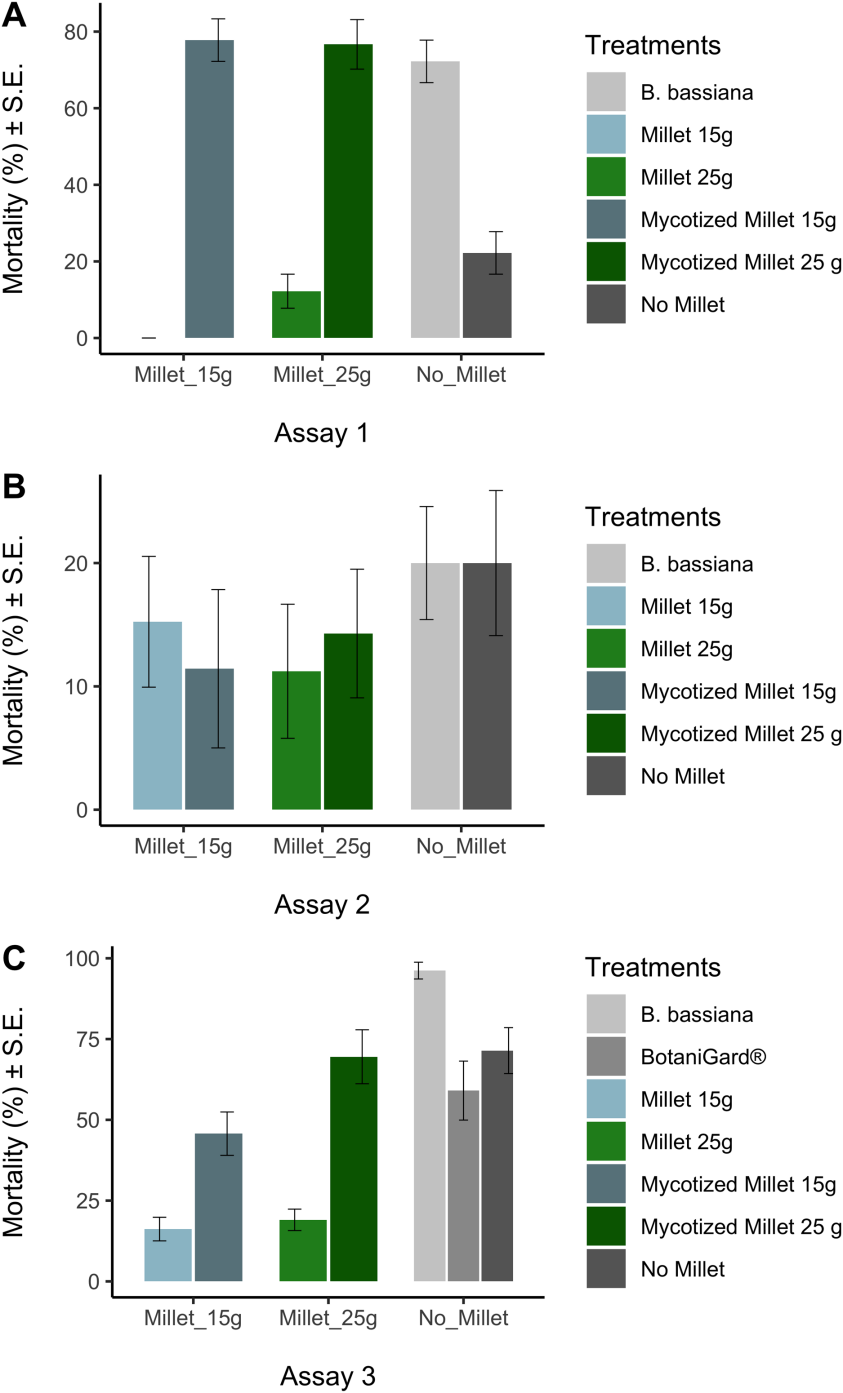
Average mortalities for controls and treatments in three bioassays. Average mortalities for controls and treatments assay 1 (A), assay 2 (B), assay 3 (C). Average mortalities and Standard Error.

Prior to analysis we used the arcsine-square-root transformation [x′ = asin(sqrt(x))] to normalize mortality data, followed by the Kolmogorov-Smirnov test (KS test) to confirm normality. The arcsin square root transformed mortality data sets for each treatment were normal at the 0.05 level. In all trials, ANOVA was used to test whether there were any differences in mortality data among treatments at the end of the experiments. Tukey’s Honestly Significant Difference (HSD) test was used to test whether the treatments were significantly different from the appropriate control. While statistics was done on arcsine-square-root transformed data, in Fig. 2 we report untransformed mortality data.

In addition, Abbott’s formula was applied to calculate mortality rates corrected for the control response (Abbott, 1925) to calculate LT_50_ and LT_99_:

(1) $$M_{corr}=\;\frac{\left\langle M_{T}\right\rangle \;-\;\left\langle M_{c}\right\rangle }{1-\;<M_{c}>}$$

where *M*_corr_ is the corrected mortality, <*M_T_*> is the average observed treatment mortality, and <*M_c_*> is the average observed mortality in the control.

The variance of *M*_corr_, Var (*M*_corr_) was calculated following Rosenheim & Hoy (1989) as

(2) $$Var\left(M_{corr}\right)=\;\frac{1}{{\left(1-\left\langle M_{c}\right\rangle \right)}^{2}}\;\left[\frac{Var\left(M_{T}\right)}{n}+{\left(\frac{1-\left\langle M_{T}\right\rangle }{1-\left\langle M_{c}\right\rangle }\right)}^{2}\frac{Var\left(M_{c}\right)}{n}\right]\;\;\;\;\;\;\left[2\right]$$

where *n* is the number of replicates, Var (*M_T_*) and Var (*M_c_*) are the variances of the treatment and control mortalities.

In the third assay mortality was recorded every 3 to 4 days starting on the second day of the experiment to calculate lethal time LT_50_ and LT_99_ assuming a logit function. These were estimated using R package Ecotox (Hlina et al., 2019).

## Results

### Sporulation and germination rates of mycotized millet grains

**Figure 1:**
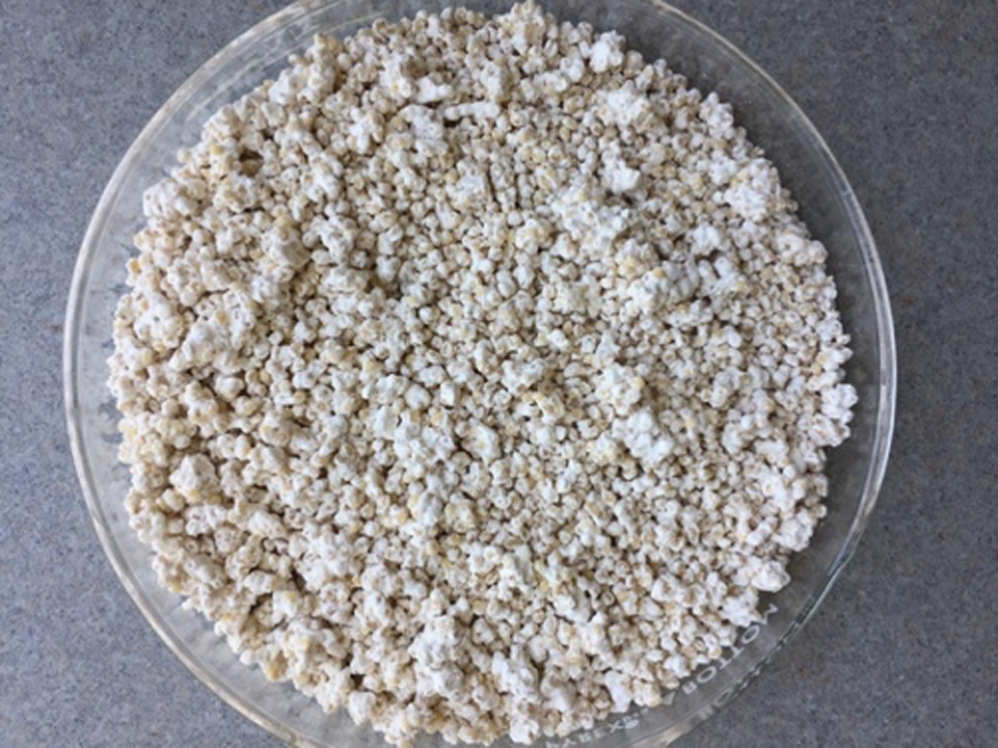
Millet grains fully mycotized by *B. bassiana* conidia. Photo credit: Maryam Nouri-Aiin.

Fungal mycelium started to grow gradually on the inoculated millet grains after 3–4 days. After 10 days all grains were fully covered by the fungus (Fig. 1). For each assay, the three bags with highest conidial production and germination rate > 98% were selected. Conidial densities per gram of grains for the selected bags for assay 1 were 3.1 × 10^8^, 1.9 × 10^8^ and 2 × 10^8^, assay 2, 2 × 10^8^, 2.1 × 10^8^ and 1.9 × 10^8^, and assay 3, 1.9 × 10^8^, 2 × 10^8^ and 2.3 × 10^8^.

### Mortality of earthworms

The Kolmogorov–Smirnov test showed the arcsine-square-root transformed distributions for each treatment were not significantly different from normal distribution. ANOVA showed that there were differences in mortality among treatments in assay 1 and assay 3, but not in assay 2 (Table 2). In assay 1, mycotized millet grain treatments were significantly different from the control millet (HSD: (*P* < 0.0001) for the 15 g mycotized millet and (*P* < 0.0001) for the 25 g mycotized milled). Likewise, the recultured *B. bassiana* treatment differed significantly from the control (*P* < 0.0001). The fungal treatments showed mortalities between 72 ± 6% and 78 ± 6%. Compared with the controls that varied between 22 ± 6% 12 ± 4% and (Fig. 2A).

**Table 2:** ANOVA table. Analysis of variance for assays.

		Df	Sum Sq	Mean Sq	*F*	*P*
Assay 1	Treatments	4	12.296	3.0739	28.55	0.001
	Error	70	7.537	0.1077		
Assay 2	Treatments	5	0.212	0.04237	0.707	0.62
	Error	83	4.97	0.059		
Assay 3	Treatments	6	15.19	2.532	19.93	0.001
	Error	98	12.45	0.127		

Assay 3 obtained similar results to assay 1 (Table 2). Comparison of millet 15 g and mycotized millet 15 g showed no significant difference (*P* = 0.079). The comparison between water and BotaniGard^®^ was also not significant (*P* = 0.939). The comparison between *B. bassiana* and water was also significant (*P* = 0.036). Comparison between millet 25 g and mycotized millet 25 g was significant (*P* < 0.0001). The recultured *B. bassiana* suspension added directly to the earthworms prior to placing them in the soil had the highest mortality (96% ± 3%) which was significantly different from all other fungal treatments (*P* < 0.043) (Fig. 2C). Lowest mortality of the fungal treatments was recorded for 15 g mycotized millet (45% ± 7%) among the treatments. However, when comparing the commercial BotaniGard^®^ treatment in this experiment, there was no statistical difference between the mortality with commercial BotaniGard^®^ (59% ± 9%) and the untreated control (71% ± 7%).

There were no significant differences in mortality in assay 2 between controls and treatments in which 1 year old product was used (Table 2). Mortalities varied between 11% and 20% (Fig. 2).

### Effect of *B. bassiana* on earthworm development

While there was no difference in mortality found for treatments in assay 2 (Table 2), there were differences in the development of the earthworms. In all millet treatments some earthworms reached maturity (Table 3), but fewer did so in mycotized millettreatments at the end of the assay (ANOVA, df = 5, *F* = 34, *P* > 0.001). There were significant differences between 15 g mycotized millet (4% ± 8%) and the 15 g millet control (40% ± 29%), and between 25 g mycotized millet (50% ± 30%) and the control with 25 g millet (73% ± 24%). The more millet, the greater the advance from juvenile to adult. And, more earthworms advanced to adult in the control treatments than the fungal treatments.

**Table 3:** Percentage of adults of assay survivors. Dissimilar letters indicates statistically significant differences for each assay.

Assay	Adult ± SE						
	Untreated control	Control millet 15 g	Control millet 25 g	BotaniGard® 10^9^ conidia/mL	*B. bassiana* 10^8^ conidia/mL	Mycotized millet 15 g	Mycotized millet 25 g
Assay 1	0%^b^	No Data	87 ± 9.08%^a^	Not Applied	0%^b^	0%^b^	0%^b^
Assay 2	0%^c^	39 ± 7.56%^b^	73 ± 6.50%^a^	Not Applied	0%^c^	4 ± 2.18%^c^	50 ± 7.99%^b^
Assay 3	20 ± 5.54%^bc^	75 ± 4.06%^a^	69 ± 6.06%^a^	27 ± 2.10%^b^	2 ± 1.09%^c^	33 ± 7.06%^b^	26 ± 2.1%^b^

In assay 1 only earthworms in the control with 25 g millet reached maturity (87% ± 35%).

In the third assay, in which juveniles were further along in their development, adults were observed in all treatments but with very significant differences among treatments (ANOVA, df = 5, *F* = 53, *P* > 0.001). Controls with 15 g and 25 g millet, not mycotized, had the largest number of adults: 75% ± 16% and 76% ± 13% respectively. In contrast, in treatments with *B. bassiana* occurrence of adults were lower: 2% ± 6% for recultured *B. bassiana*, 15 g mycotized millet 33% ± 27% for 15 g of mycotized millet, and 26% ± 28%, for the 25 g mycotized millet treatment. The untreated control scored a similar development rate to the fungal treatments (20% ± 21%).

LT_50_ and LT_99_ values calculated with Abbott’s correction are listed in Table 4. The shortest LT_50_ and LT_90_ were observed for the recultured *B. bassiana* suspension added directly on earthworms (16 days and 45 days respectively). In contrast, lethal times were longer for BotaniGard^®^ (30.2 and 76.1 days). The more mycotized millet is added, the shorter the lethal times.

**Table 4:** Treatment Lethal time (days) calculated for Abbott corrected mortalities. Treatment Lethal times (days), LT_50_ and LT_99_, calculated for each treatment used in assay 3. Upper and lower confidence limits (CL) are shown in parentheses.

	Lethal Time Logit	
Treatments	LT_50_ (95% CL)	LT_99_ (95% CL)
*B. bassiana*	16 [13.5–18.4]	45 [38.0–57.8]
Mycotized millet 15 g	35.4 [32.4–40.0]	74.1 [63.7–91.1]
Mycotized millet 25 g	21.8 [18.7–26.2]	66.2 [52.7–96.5]
BotaniGard^®^	30.2 [24.5–49.5]	76.1 [54.0–178.0]

## Discussion

Entomopathogens have not been considered as options for controlling invasive earthworms. As the name suggests entomopathogens are known to infect insects which have a different physiology and anatomy to earthworms. Earthworms are also known for their robust immune system (Prochazkova et al., 2019). And it is possible that they can defend against fungal pathogens. To our knowledge, there has only been one study with earthworms that found mortality and weight loss in *E. foetida* caused by fungi, specifically *Aspergillus* spp., *Penicillium* spp. and *Trichoderma* spp. (Edwards & Fletcher, 1988).

There are many factors involved in the efficacy of an entomopathogen on the host such as host defense mechanism, biotic and abiotic factors, and efficacy of the fungal strain (Vega et al., 2009; Shahid et al., 2012). In this study the age of the product, from which a pure culture of *B. bassiana* was cultured, affected outcomes of one the assays. The mortality rate was higher in assay 1 and 3 when fresh conidia were used than in assay 2 when 1-year-old product was used. However, there was still an effect of the older product on the development of the earthworms. Prolonged storage of *B. bassiana* under 20 °C did not cause loss of virulence (Sandhu, Rajak & Agarwal, 1993). However, in this study we worked with a commercial product while Sandhu, Rajak & Agarwal (1993) worked with pure *B. bassiana* culture with no inert ingredient in them which might have affected the virulence or longevity of the active ingredient. Their trials showed the age of the product had no effect on the conidial germination rate as was proposed by Le Grand & Cliquet (2013). Although the pathogenicity of the fungus was affected by the age of the product, the culture derived from older conidia still had an effect. Specifically, the older spores retarded the advance of the earthworms from juvenile to adult. The pheretimoid species produce about one cocoon every other day per worm at the same study site that we collected the earthworms for the assays (Nouri-Aiin & Görres, 2019). Shortening their reproductively active period by delaying the reproductive stage would probably result in fewer offspring the following year.

The life stage of the earthworms might have also influenced the outcome of this study. Pheretimoid juveniles collected in June were more susceptible to both *B. bassiana* suspension and mycotized millet (assay 1) than pre-adult juveniles collected in late August (assay 3). Untreated millet accelerated maturation of these earthworms. This may be of interest to those who want to grow experimental populations of pheretimoids.

LT_50_ values in this experiment with earthworms were longer than observed in insect larvae subjected to this control agent. The Pheretimoid earthworm LT_50_ was between 13 and 22 days. Butt et al. (1994) reported LT_50_ values between 4 and 14 days for crucifer pests with the longer LT_50_ value associated with conidial concentrations two order of magnitudes lower than ours. Akmal et al. (2013) reported LT_50_ for different aphid species of around 3 days. LT_90_ values in this experiment were about twice as long as reported by Butt et al. (1994). In comparison with other studies on insect mortality with *B. bassiana* (e.g., Bugeme et al., 2008; Ozdemir et al., 2020), LT_90_ was also much longer for pheretimoids. Some experiments are reported in the literatures that evaluated the effect of pesticides on earthworm’s mortality, albeit on different species than those in this study. For example, Potter et al. (1990) studied effect of 17 commonly used turfgrass pesticides on earthworms. They reported 2.6–99.0% mortality in first week of pesticide application which is much shorter than *B. bassiana*.

The three most important pheretimoid invaders are annual earthworms which require about 90 days from hatchling to maturity (Görres & Melnichuk, 2012). However, hatching occurs year-round (Görres et al., 2018; Nouri-Aiin & Görres, 2019) and peak abundance is usually achieved in late May or early June in Vermont, this may vary with climate zone, and the rate of accumulation of heat unit (Görres, Bellitürk & Melnichuk, 2016). First adults are observed in early June to mid-August (Görres, Bellitürk & Melnichuk, 2016; Nouri-Aiin & Görres, 2019). Considering these life history traits with LT_90_s, several applications of *B. bassiana* would need to be planned to successfully control invasive populations.

In this experiment the commercial formulation of *B. bassiana* was not as effective as the re-cultured product. There may be several reasons for this. Fillers in the product may interfere with the efficacy when applied to earthworms. Another would have to do with mechanism by which *B. bassiana* acts. There are several stages from infection to the death of insects. Infection in insects occurs on contact between the insect and the fungus. Germination tubes of conidia penetrate the cuticle. Then, the fungus grows and produces an array of entomotoxins which subsequently kill the insect. It is possible that in our experiments the toxin is produced during culturing the commercial product and that when applied to earthworms it is the residual toxin in the culture that kills the earthworm. However, this does not explain the length of time it takes to kill earthworms (LT_50_ ~ 20 days). It is possible that the innate immune response of earthworms can resist the fungus and slow the infection (Bilej et al., 2010). The celomic fluid of earthworms contains many potential pathogens that are ingested from their natural habitat. The earthworm immune system is built to resist these pathogens through phagocytes that consume the pathogens (Dales & Kalaç, 1992). Earthworms also produce antimicrobial compounds (Meghvansi et al., 2011; Vasanthi, Chairman & Singh, 2013) that may reduce the chance of infection. We did not verify the cause of death by culturing the fungus from the deceased earthworms, as they decompose rapidly.

Mycotized millet was added to the soil was as effective as the recultured suspension sprayed directly on the earthworms. Mycotized millet has two potential functions that make it effective. Because of *B. bassiana* saprophytic nature, millet provides a good substrate for it to stay viable for longer in soil (Hua & Feng, 2005; Saranraj & Javaprakash, 2017). It also may act as bait for the earthworms and thus facilitates contact between earthworm and fungus. In the millet controls, pheretimoids were more likely to become adults over the incubation period and their survival was superior to the other treatments including the control where just water was added. This suggests that millet was a food source utilized by these earthworms. Of course, the downside of using mycotized millet is that it is not commercially produced.

Immediate questions for future research arising from this study are: Why does the recultured formulation perform better than the commercial product from which it was derived? What causes the long LT_50_? Are there other pathogens that can kill these invasive forest pests faster?

This study did not address how *B. bassiana* might affect the viability of cocoons. These are not only reproductive, but also survival structures and may afford the embryos some protection from the fungus. The one study that evaluated the effect of entomopathogenic fungi on cocoons was not conclusive (Nuutinen et al., 1991) and more studies specifically on cocoon viability are.

## Conclusions

Our results suggest that using *B. bassiana* can be considered as a potential biocontrol agent to prevent the spread of invasive pheretimoids from greenhouses and nurseries into forest ecosystems. However, its mode of action causing the morbidity of earthworms is not clear and further studies are needed.

## Supplemental Information

10.7717/peerj.11101/supp-1 Supplemental Information 1 Raw data. Bioassay 1, 2, and 3 results.

## Additional Information and Declarations

### Data Availability

The following information was supplied regarding data availability: Survival and mortality data for pheretimoid earthworms subjected to *Beauveria bassiana* are available as a Supplemental File.
